# Safety of the Fiocruz ChAdOx COVID-19 vaccine used in a mass vaccination campaign in Botucatu, Brazil

**DOI:** 10.1016/j.vaccine.2022.08.026

**Published:** 2022-11-08

**Authors:** Sue Ann Costa Clemens, Carlos Magno Castelo Branco Fortaleza, Madeleine Crowe, Andrew Pollard, Karen Ingrid Tasca, Rejane Maria Tommasini Grotto, Marcelo Roberto Martins, André Gasparini Spadaro, Pasqual Barretti, Tom Verstraeten, Ralf Clemens

**Affiliations:** a Department of Pediatrics, Oxford University, Oxford, United Kingdom; b Institute for Global Health, Siena University, Siena, Italy; c Department of Infectious Diseases, Botucatu Medical School, São Paulo State University (UNESP), City of Botucatu, São Paulo State, Brazil; d P95 Epidemiology & Pharmacovigilance, Leuven, Belgium; e Department of Biotechnology, Faculty of Agronomical Sciences, São Paulo State University (UNESP), City of Botucatu, São Paulo State, Brazil; f Division of Informatics, Botucatu Medical Hospital, Botucatu Medical School, São Paulo State University (UNESP), City of Botucatu, São Paulo State, Brazil; g Botucatu Health Department, City of Botucatu, São Paulo State, Brazil; h Department of Clinical Medicine, Botucatu Medical School, São Paulo State University (UNESP), City of Botucatu, São Paulo State, Brazil; i International Vaccine Institute (IVI), Seoul, South Korea

**Keywords:** COVID-19, ChadOx1-nCoV19, Adverse events, Vaccine safety, Immunisation

## Abstract

**Introduction:**

Brazil has been at the core of the COVID-19 pandemic, with the second-highest death toll worldwide. A mass vaccination campaign was initiated on May 16th, 2021, in Botucatu, Brazil, where two doses of ChadOx1-nCoV19 were offered 12 weeks apart to all 18–60- year-olds. This context offers a unique opportunity to study the vaccine safety during a mass campaign.

**Methods:**

The first and second doses of the vaccine were administered in May and August 2021, respectively. Emergency room (ER) and hospitalization records were obtained from the Hospital das Clínicas da Faculdade de Medicina de Botucatu for six weeks before and six weeks after the first and second doses, from 4 April to 19 September 2021. Diagnoses with COVID-19-related ICD codes were excluded to distinguish any trends resulting from the COVID-19 pandemic. ER and hospital visits during the two time periods were compared, including an ICD code comparison, to identify any changes in disease distributions. Data were scanned for a defined list of Adverse Events of Special Interest (AESIs), as presented by the Safety Platform for Emergency Vaccines.

**Results and discussion:**

A total of 77,683 and 74,051 subjects received dose 1 and dose 2 of ChadOx1-nCoV19, respectively. Vaccination was well tolerated and not associated with any major safety concerns. Increases in ER visits 1 week following both doses were primarily seen in ICD codes related to non-serious side effects of the vaccine, including vaccination site pain and other local events. The neurological AESIs identified (2 of 3 cases of multiple sclerosis) were relapses of a pre-existing condition. One potentially serious hospitalization event for Bell’s palsy had onset before vaccination with dose 1, in a patient who also had a viral infection of the central nervous system. There was no myocarditis, pericarditis cases, or vaccine-related increases in thromboembolic events.

## 1 Introduction

Brazil has been at the core of the COVID-19 pandemic, reporting the second highest death toll worldwide [1]. The autonomy of municipality and state governments has led to varying policies and degrees of implementation of non-pharmacological interventions (NPIs), including the use of masks and mobility restrictions across the country [2], [3], [4]. As these measures have had a varying impact [5], interventions such as mass immunization can be an important tool to rapidly contain the spread of SARS-Cov-2 [6].

At the time of the project, two vaccines were primarily used in the national immunization program (NIP). These were Coronavac®, developed by the Chinese company Sinovac in partnership with the Butantan Institute [7], and ChadOx1-nCoV19® (registered in Brazil as “recombinant COVID-19 vaccine”), developed by the University of Oxford and AstraZeneca, produced and distributed in Brazil by the Oswaldo Cruz Foundation (FioCruz) [8].

Mass vaccination offers a unique opportunity to study the safety of the intervention by comparing disease rates immediately before and after the campaign. We took advantage of a pre-planned study of the effectiveness of the recombinant COVID-19 vaccine from Oxford/Fiocruz in a large-scale administration to also assess the safety of this vaccine.

## 2 Methods

### 2.1 Study population and design

We conducted an ecological study looking at event rates before and after the mass vaccination campaign conducted in Botucatu, Brazil, a city with a population of 142,092 inhabitants. Botucatu is a city in inner São Paulo State, harboring a university hospital that provides tertiary care for surrounding municipalities, an area comprising half a million people. The municipal health department adheres to the family health program within Brazil's socialized Unified Health System (SUS); and historically has had high adherence to previous vaccination campaigns.

All citizens aged 18–60 years (N = 92,349) were eligible for inclusion in the campaign independent of any underlying condition. Vaccination was offered by qualified healthcare workers in 45 election-voting locations and four school courts with which the vaccinees were familiar, to facilitate campaign adherence. A total of 65,450 citizens aged 18–60 years received a first dose of ChadOx1-nCoV19 on a single day in the campaign, Sunday, May 16. Another 12,233 were vaccinated in the following four weeks, resulting in a total of 77,683 people receiving a first dose of the vaccine. 60,333 vaccinees received their second ChadOx1-nCoV19 dose 12 weeks later on Sunday, August 8, with an additional 13,718 citizens over the next four weeks for a total of 74,051 s doses. Thus, vaccination coverage of the 18–60-year-old population of Botucatu was 84.2% and 80.2% for the first dose and second dose, respectively. The main objective of the larger project was to determine the overall and strain-specific vaccine effectiveness in a mass campaign setting.

A secondary objective was vaccination safety. We obtained ICD-10 coded hospital and ER diagnoses from the Hospital das Clínicas da Faculdade de Medicina de Botucatu in Botucatu, Brazil, for patients of all ages resident in Botucatu, from six weeks before to six weeks after the first and second doses spanning the period from 4 April to 19 September 2021. The hospital covers 80% of the Botucatu population for all ER visits. Confirmed, suspected and probable COVID-19 cases (coded as U071, U072, and B342), as well as other respiratory infections (codes B349, J0-J2s) as documented in the database, were excluded from the analysis to distinguish the vaccination safety findings from any clinical events resulting from the ongoing COVID-19 pandemic. As the campaign targeted adults 18–60 years old, we focused our analyses on patients in this age group (referred to as the “vaccinated” cohort). All ER and hospital visits were counted, except in the case that a patient attended the healthcare facility more than once in a given day, in which case the earliest record was taken as presented in the dataset.

### 2.2 Outcomes

We looked at three different outcomes. To identify any increases in ER or hospitalization rates, we compared the overall ER visit and hospitalization rates on a weekly basis in the six weeks preceding the campaign to six weeks after dose 1 and dose 2. May 16 and August 08 were taken as “day 0” for dose 1 and dose 2 respectively. To identify any increase in specific causes of hospitalization or ER visits, we compared the distribution of ICD codes before (otherwise referred to as baseline) and after mass vaccination. As a final step, records in the vaccinated cohort were scanned for a defined list of Adverse Events of Special Interest (AESIs), as presented in the Safety Platform for Emergency Vaccines (SPEAC); this list was expanded to include other potential serious events based on recent safety reports of COVID-19 vaccines such as myocarditis/pericarditis and Bell’s palsy. Additional information about these cases was sought individually to understand potential links to vaccination, including cross-checking information on actual vaccination status and date of vaccination. We counted AESI by individual. If one AESI was reported both before and after vaccination, this was counted in the before category, and only the most serious event was noted in that category. Although the catchment area for the clinic included cities outside of Botucatu, analyses were restricted to patients from Botucatu.

### 2.3 Statistical analysis

ER and hospitalization rates were calculated as $\frac{\mathrm{numberofERorhospitalvisits}}{\mathrm{timeperiod}}$, whereby the time period corresponded to the period of the study, i.e. 42 days. We assumed that the population denominator would be stable over the study period and, therefore, did not use this parameter in our calculations. To assess any increase in rates of AESIs we simply compared the absolute number of such events as we used similar lengths of observation time (six weeks before and after each dose) and assumed again that the population would be stable in this period. All results reported in this study were descriptive.

We compared the age/gender distribution in the population aged 20–60 years of age in Botucatu [9], with the population that attended our healthcare facilities in order to highlight any potential differences.

## 3 Results

### 3.1 ER and hospitalization rates

A total of 20,769 ER and 2,394 hospitalization diagnoses were reported among patients aged 18–60 years old residing in Botucatu during the period from April 4 to 19 September 2021, including multiple ER visits and multiple hospitalization diagnoses for the same patients. We noted a higher percentage of females in the 20–24, 25–29, and 30–34 years of age categories when compared to the overall age/gender distribution in Botucatu (see Table 1).

**Table 1:** Age gender distribution, population of Botucatu vs those seen at our healthcare facilities, 20–60 years old.

Age Group (years old)	Total by age group (%)		Gender			
			Female (%)		Male (%)	
	Botucatu	HCF	Botucatu	HCF	Botucatu	HCF
20–24	10,221 (12.2)	4439 (16.3)	5048 (49.4)	2785 (62.7)*	5173 (50.6)	1654 (37.3)*
25–29	11,307 (13.5)	4032 (14.8)	5631 (49.8)	2596 (64.4)*	5676 (50.2)	1436 (35.6)*
30–34	11,862 (14.2)	3686 (13.5)	5917 (49.9)	2242 (60.8)*	5945 (50.1)	1444 (39.2)*
35–39	12,008 (14.3)	3685 (13.5)	6087 (50.7)	2155 (58.5)	5921 (49.3)	1530 (41.5)
40–44	11,302 (13.5)	3436 (12.6)	5773 (51.1)	1770 (51.5)	5529 (48.9)	1666 (48.5)
44–49	9961 (11.9)	3125 (11.5)	5110 (51.3)	1660 (53.1)	4851 (48.7)	1465 (46.9)
50–54	8859 (10.6)	2424 (8.9)	4642 (52.4)	1223 (50.5)	4217 (47.6)	1201 (49.5)
55–59	8251 (9.8)	2434 (8.9)	4390 (53.2)	1189 (48.8)	3861 (46.8)	1245 (51.2)

*More than a 10-percentage point difference between the age/gender distribution in Botucatu vs attending our healthcare facilities.

### 3.2 Dose 1

ER and hospital visits showed an increasing trend starting prior to the administration of dose 1 of the mass vaccination campaign, with a slightly higher rate of visits in the six weeks after vaccination, when compared to the pre-vaccination period (Fig. 1a). The average weekly number of ER visits was 788 at baseline and 887 after the campaign (a 12.6% increase), and the average weekly number of hospitalizations was 99 at baseline and 105 after the campaign (a 6.1% increase). Specifically for ER visits, a steadily increasing trend prior to vaccination was observed, with a peak in week 1 after vaccination. Hospitalizations showed a slower increasing trend, up until one week prior to vaccination, with no increase in hospitalizations in week 1 after vaccination.

**Fig. 1a:**
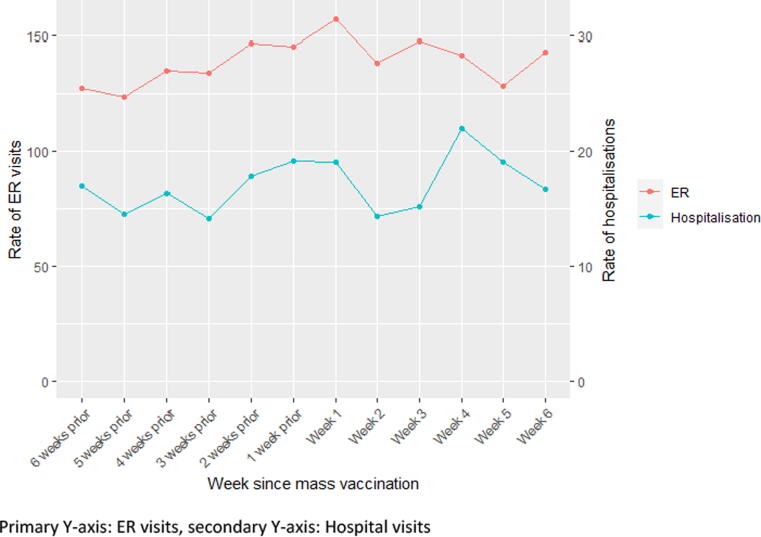
Weekly rate of visits-Post dose 1.

### 3.3 Dose 2

A clear increase in ER visits was observed in the six weeks after dose 2 compared to the prior six weeks, with an average weekly number of visits of 782 before and 1004 following the campaign. This increase continued throughout the entire six weeks after dose 2. Hospital visits showed the opposite trend, with a lower average number of hospitalizations in the six weeks after dose 2 compared to the prior (95 compared to 100). The increase in the first week after dose 2 was much more prominent than that observed in the first week after dose 1 (see Fig. 1b).

**Fig. 1b:**
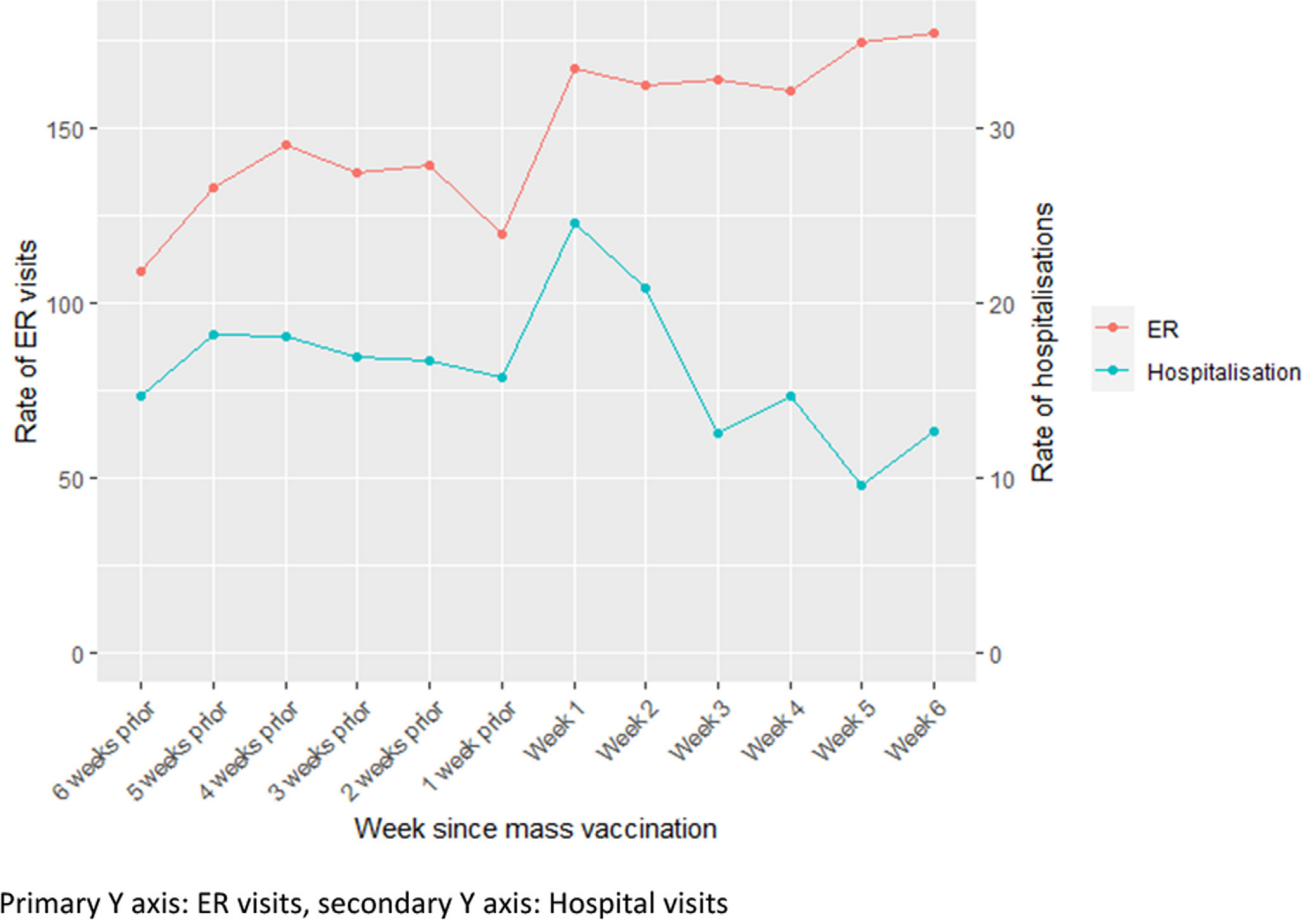
Weekly rate of visits- Post dose 2.

### 3.4 ICD distributions

The distribution of ICD codes by category was not different in a clinically relevant way when comparing the two time periods (after dose 1 and after dose 2, Figs. 2a and 2b). However, a few categories showed higher weekly counts, most notably the R, T, Y and Z subgroups.

**Fig. 2a:**
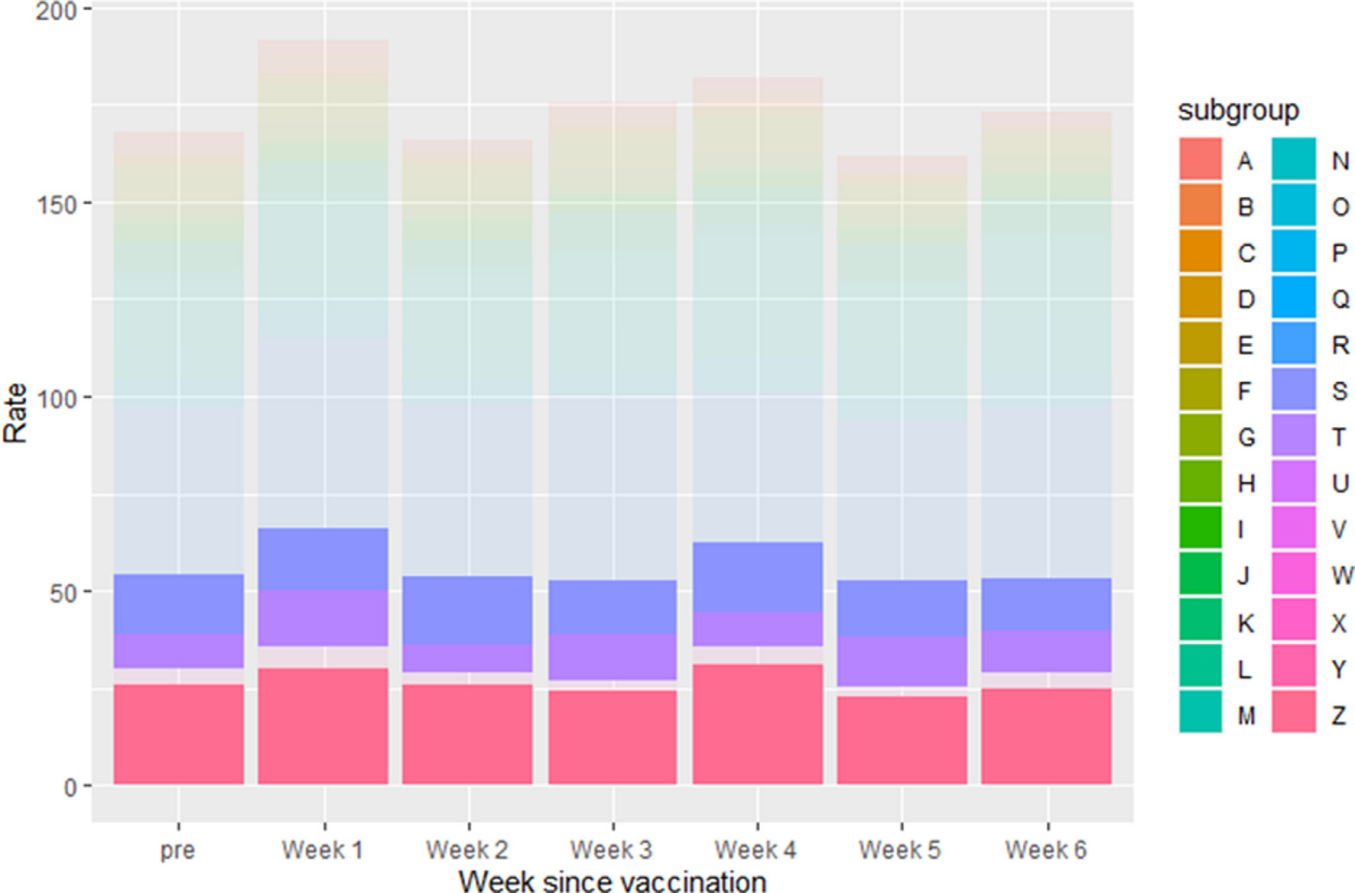
ICD subgroups over time- Post dose 1.

**Fig. 2b:**
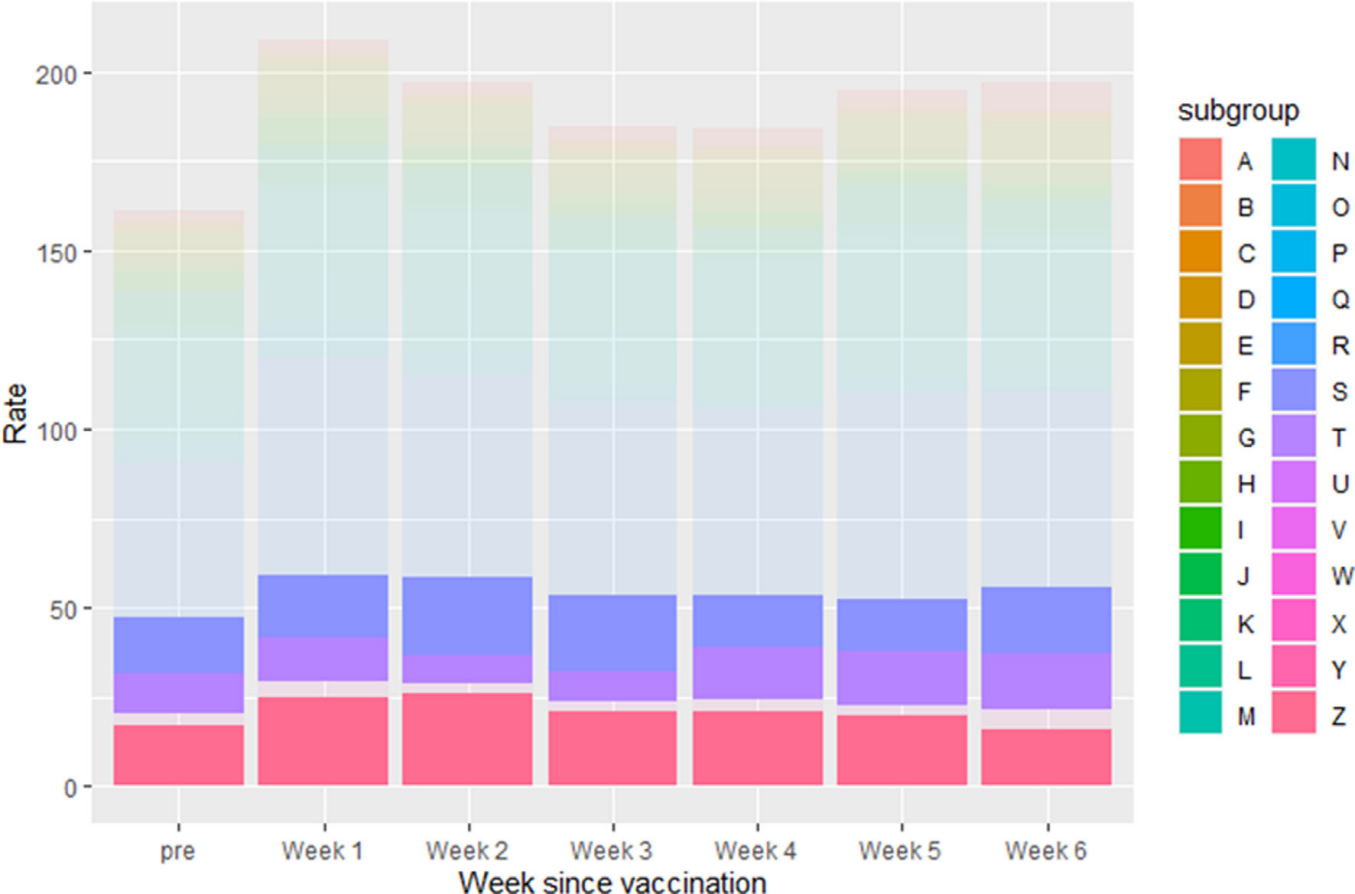
ICD subgroups over time- Post dose 2.

### 3.5 Dose 1

The highest relative increase in ICD codes from baseline to week 1 after vaccination was observed in the Y subgroup, a group consisting of external causes of morbidity and mortality, with events related to “adverse reactions to vaccines” (2.9 times the baseline, Y590; Y598-99).No distinction could be made on which adverse reactions these were. In terms of absolute numbers, the largest differences from baseline to week 1 after vaccination were seen in the “R” group, followed by “T” and “Z”. Within the R category, consisting of symptoms, signs and abnormal clinical and laboratory findings, not elsewhere classified, unspecified pain (R520) contributed the largest proportion, followed by malaise and fatigue (R53). The “T” subgroup consists of categories related to injuries; of which the majority identified were unspecified, multiple injuries (T07). Finally, the “Z” subgroup comprises factors influencing health status and contact with health services, of which general adult examination (Z000) and supervision of normal pregnancy (Z349) were the codes that had the largest increase between the two periods.

### 3.6 Dose 2

Following the administration of dose 2, similar increases in ICD codes were seen, with the addition of headache (R51) and supervision of high-risk pregnancy (Z359) contributing the highest absolute increases.

### 3.7 AESIs

There were 18 predefined AESIs assessed in this study. No cases were reported at any time for six of the 18 AESIs, including Parkinson’s disease, Guillain-Barré Syndrome, (meningo-)encephalitis, optic neuritis, and myocarditis. For 7 AESIs, the number of reports was comparable in the period before and after vaccination, including convulsions, thrombocytopenia, hemorrhagic conditions, pulmonary embolisms, arterial embolisms, anaphylactic shock, vasculitis, and pericarditis. For one AESI (pulmonary embolism), the number of events reported decreased from seven before vaccination to two after vaccination. Finally, an increased number of cases occurred for four of the 18 AESIs: Bell’s palsy (after dose 1), demyelinating disorders (after dose 2), venous thrombosis (after dose 2), and unspecified allergic reactions (after dose 2) (Table 2). These reports are discussed in more detail.

**Table 2:** Hospitalisations by AESIs.

Class	Condition (ICD-10 code)	Number ER visits (hospitalisations)			
		Dose 1		Dose 2	
		Pre-mass vaccination	Post-mass vaccination	Pre-mass vaccination	Post-mass vaccination
Neurological	Parkinsons (G200);	0 (0)	0 (0)	0 (0)	0 (0)
	MS, other demylinating diseases (G35; G37);	0 (0)	0 (0)	0 (0)	3 (0)
	Guillain-Barré Syndrome (G61);	0 (0)	0 (0)	0 (0)	0 (0)
	Meningoencephalitis (B4081; G042; A390; A394);	0 (0)	0 (0)	0 (0)	0 (0)
	Encephalitis; inc acute disseminated encephalomyelitis (G04s),	0 (0)	0 (0)	0 (0)	0 (0)
	Optic neuritis (H46), Neuromyelitis optica (G36)	0 (0)	0 (0)	0 (0)	0 (0)
	Bell’s Palsy (G51.0)	1 (0)	4 (1)	1 (0)	1 (0)
	Generalised convulsions (R568) incl. epilepsy (G40s)	25 (9)	28 (7)	14 (3)	16 (4)
Hematological	Thrombocytopenia (D696), immune thrombocytopenic purpura (D693)	0 (0)	0 (0)	1 (0)	0 (0)
	Hemorrhagic condition, unspecified (D699)	1 (0)	0 (0)	0 (0)	1 (0)
	Pulmonary embolism with/without acute cor pulmonale (I260; I269)	0 (3)	0 (1)	0 (4)^1^	0 (0)
	Embolism and thrombosis of the veins (I828; I829)	2 (1)	3 (0)	3 (2)^2^	6 (0)^3^
	Embolism and thrombosis of the arteries (I742; I743; I748; I749)	1 (1)	0 (1)	2 (2)^4^	3 (1)
Immunologic	Anaphylactic shock, unspecified (T782)	0 (1)	0 (0)	0 (0)	1 (0)
	Other unspecified allergies (T784)	27 (0)	31 (0)	21 (0)	40 (0)^5^
	Vasculitis (L51.9; L959)	0 (0)	0 (0)	0 (0)	1 (0)
Cardiovascular	Myocarditis ((I40.9; I40.8; I40.0;I40.1)	0 (0)	0 (0)	0 (0)	0 (0)
	Pericarditis (I30.1; I30.8-9)	0 (0)	0 (0)	0 (1)	0 (1)

1 One of these cases had only received one dose of ChadOx1-nCoV19, one had not been vaccinated.

2 1 of 2 hospitalisations had received no vaccine, and 2 of 3 ER visits had received one dose of BNT162b2. The remaining had only received one dose of ChadOx1-nCoV19 (1 hospitalisation and 1 ER visit).

3 Two of these 6 cases had only received one dose of the ChadOx1-nCoV19.

4 1 of 2 ER visits and both hospitalisations had only received one dose of ChadOx1-nCoV19. The other ER visit had received two doses of Coronavac.

5 5 of 40 had received another type of vaccine (Coronovac), 2 of 40 only received one dose of ChadOx1-nCoV19, 2 of 40 were not vaccinated at all, and 1 of 40 had no available information on vaccination status.

### 3.8 Bell’s palsy

Eight cases of Bell’s palsy were reported in total during the study period, two during the pre-vaccination period, five after dose 1 (in three females aged 24–50 years old, and two males aged 50–55 years of age) and one after dose 2 (female aged 25 years of age). Five of these six cases after vaccination were reported between weeks 3 and 6 after vaccination: however, one had had onset of symptoms prior to vaccination but was reported late, and another patient with Bell’s palsy had a previous documented COVID-19 infection (M55). A third patient had been previously diagnosed with stroke (I64), had onset of Bell’s palsy symptoms prior to being vaccinated with ChadOx1-nCoV19, and was hospitalized one day after vaccination for ten days. He was concurrently diagnosed with a viral infection of the central nervous system and recovered with sequelae (M50).

### 3.9 Demyelinating disorders

Two cases of MS were reported in weeks 2 and 3 after dose 2. Both events were relapses of pre-existing MS in females aged 28 and 58 years old. A third case of demyelinating disease of unknown origin in a male aged 26 years old was reported, identified as either a possible after-vaccine monophasic disease or a first outbreak of multiple sclerosis; with the onset of symptoms starting prior to the administration of dose 2. Cough and rhinorrhea at the onset of symptoms were noted, suggestive of a viral infection, but no further virological work-up was done.

### 3.10 Venous thrombosis

There were three venous thrombosis events in the six weeks observation period prior to vaccination and also the same number in the six weeks after dose 1. Seven venous thrombotic events were recorded after dose 2, in three females aged 21–47-year-old, and 5 males aged 47–51 years old. Of those, two patients had had a previous diagnosis of deep vein thrombosis (DVT, M51, F47) and two others developed a DVT following hospitalization for other purposes (appendectomy (M47) and a cancer diagnosis (M51)). The fifth patient had several underlying risk conditions for thrombotic events such as atrial fibrillation, mitral stenosis, and polyglobulia (M49). He was hospitalized and underwent a thrombectomy and eventually passed away. The venous thrombosis of the sixth and seventh patient appeared to be linked to COVID-19 infection; the patient reported symptoms of DVT following a COVID-19 diagnosis, which had occurred 5 weeks prior, with acute flare-ups of the condition after vaccination (F36, F21).

### 3.11 Allergic reactions

An almost doubling in the number of unspecified allergies was reported, from 21 to 40 in the six-week period after dose 2. Of these 40 cases after dose 2, only two (5%) were potentially related to the vaccine. Other cases were considered unrelated based on timing (before vaccination, or several weeks after); receipt of other vaccines and/or medications; or allergies based on insect bites, food, medications or contact dermatitis.

## 4 Discussion

We took advantage of a COVID-19 mass vaccination campaign targeting all adults of an entire municipality in Brazil to monitor the safety of the ChadOx1-nCoV19 vaccine used in the campaign. We looked for changes in the overall ER attendance and hospitalization rates, the reasons for these hospitalizations and a selection of adverse events of special interest. We noted a number of changes; most of which can be attributed to causes other than the vaccine. We observed an increase in the number of ER visits following the vaccination campaign, which was most notable following dose 2, compared to the period before the campaign. In the case of dose 1, this increase continued from a trend that had started before the campaign. The number of hospitalizations increased in the first two weeks after dose 2 but rapidly declined after these initial weeks.

We looked at the overall age and gender distribution of the population of Botucatu compared to the distributions that attended our healthcare facility. We noted a larger proportion of females in the age groups 20–24, 25–29, and 30–34 years of age compared to the overall distribution reported in Botucatu. This may reflect the differential healthcare seeking behaviors between males and females, as females are more likely to seek healthcare than males [10].

The analysis of ICD code distribution between baseline and week 1 following vaccination after both doses indicated a relative increase in the ICD “Y” subgroup and absolute increases in the subgroups “R”, “T”, and “Z” following the vaccination campaign. Absolute or relative increases in these subgroups, especially in “R” and “T” subgroups can be anticipated: most of those are to be considered as non-serious side effects of the vaccine: vaccination site pain (“unspecified pain”), malaise and fatigue, or incorrect vaccine administration [11]. The “Z” subgroup indicated an increased contact with healthcare providers. This was triggered by an increase in prenatal routine healthcare visits without any disease or symptomatology. Among the predefined AESIs, we observed either no or a similar number of cases being reported both before and after the vaccination campaign, with the exception of Bell’s palsy, demyelinating diseases, venous thrombosis, and unspecified allergies.

The expected incidence of Bell’s palsy globally is estimated at 15–30 per 100 000 person-years [12]. Applying this rate to the combined after-vaccination periods, we would expect 2–5 case reports just by chance, meaning that our results fall just outside of this upper bound. Although various studies reporting cases of Bell’s palsy following vaccination have been described in the literature [13], [14], [15], [16], there is still no definitive association of the link between COVID-19 vaccination and Bell’s palsy [14]. One such study conducted in Hong Kong looking at the age-standardized incidence of Bell’s palsy following vaccination compared with background rates in 2020 indicated an additional 2.0 [following vaccination with BNT162b2] and 4.8 [following vaccination with CoronaVac] cases of Bell’s palsy per 100 000 persons within 42 days of receiving each vaccine. A subsequent nested-case control study noted a significantly increased risk of developing Bell’s palsy after CoronaVac (OR 2.86), however, not after BNT162b2 [15].

An initial assessment report by the European Medicines Agency (EMA) of the ChadOx1-nCoV19 vaccine efficacy trials found no imbalance of cases of facial paralysis in the vaccinated and control groups, reporting one case in the vaccinated arm whereby causality to the vaccine could not be excluded [11]. Currently, the UK Medicines and Healthcare products Regulatory Agency (MHRA) suggests in its review of yellow card reports (up until 13 January 2022) that the incidence of Bell’s palsy is currently similar to the natural rate and does not suggest an increased risk following vaccination against COVID-19 [17].

Of note, recent studies reporting on the safety of the mass vaccination campaigns conducted in Israel and the United Kingdom noted increases in several AESIs both following natural infection with COVID-19 and following vaccination [with BNT162b2 [13] and/or ChadOx1-nCoV19 [16]]**;** making it difficult to establish whether the events found in our study were related to vaccination or to the ongoing pandemic. Both studies noted elevated risks of Bell’s palsy following vaccination (risk ratio of 1.32 following vaccination with BNT162b2, and on the day of exposure, an incidence rate ratio (IRR) for hospital admission or death of 0.33 after ChadOx1-nCoV19 vs 33.23 when compared with natural infection), suggesting that vaccination may have a stronger association with Bell’s palsy than natural infection. Higher risk ratios of developing DVT following natural infection when compared with vaccination (risk ratio of approximately 0.9 versus 3.1 after natural infection) were also noted [13], and currently, there is no evidence of an association between DVT and COVID-19 vaccination in the pharmacovigilance sphere [17].

Relapses of MS or acute CNS demyelinating events following natural infection with COVID-19 and with vaccination have been reported in the literature [16], [18], [19]; however, no significant short-term increases following either have been noted. Patone et al. reported no association in any 7-day risk period following vaccination with BNT162b2 or ChadOx1-nCoV19, however, on the day of a positive SARS-CoV-2 test, found an IRR of 19.34 [16]. Interestingly, in the 1–28 days after exposure [to any vaccine or positive SARS-Cov-2 test], non-significant IRRs and hence no association was found, indicating that the risk for hospitalization following vaccination or SARS-CoV-2 infection may occur in a time-dependent manner [16].

The findings in our study corroborate the global epidemiology of MS; the prevalence is higher in females in general [20] and, in relation to relapses, the age-attenuation of MS relapses is delayed in females when compared to males [21]. These findings highlight the need to monitor patients with pre-existing conditions that could be exacerbated by vaccination. Accordingly, the EMA has recently added transverse myelitis, a rare neurological condition, to the list of adverse drug reactions [22].

Our study is subject to a number of limitations. Hospitalizations that were recorded in the first week following mass vaccination may have been related to illnesses that had started in the week prior, which would have artificially been linked to vaccination. We also assumed that the healthcare-seeking behavior would not change in Botucatu after the implementation of the mass vaccination campaign. However, the analysis of the most frequent diagnoses or interventions showed increases for events not typically expected to be related to vaccination, such as general examinations and antenatal care. It is very likely that healthcare seeking increased as the population felt “safer” and more willing to go to healthcare facilities; however, further research by social scientists is warranted to explain this observation. It is however also possible that if the “after” periods coincided with the peak of the pandemic, we would have missed cases that would have normally sought care in the absence of the pandemic. We also assumed that the population remained stable throughout the study period, however as complete follow-up information about the population was not available, our rate estimates may have been affected. To the best of our knowledge, there was no significant factor which would have led to a change in the study population.

Although the vast majority of people 18–60 years of age were vaccinated on the 16th of May 2021 and the 8th of August 2021, a small percentage (9.3%) were vaccinated prior to this date. This could lead to an underestimation of the reported AESIs after vaccination; however, as the total number of AESIs both before and after vaccination was relatively low and in similar numbers, it is unlikely that we would have missed any important changes.

AESIs were classified according to timing since vaccination after each dose offered during mass vaccination; either “before” or “after”. For some conditions, however, those classified as “before” dose 2 could potentially still be linked to dose 1 and were acknowledged as such at the analysis and discussion phase. Because of the ecological nature of this study and the fact that we only sought individual vaccination status of all persons where there was a doubling in any AESI event, reporting may include events that occurred in individuals who only received one dose but in which the event occurred outside the six-week period after dose 1 or who had received a vaccine other than ChadOx1-nCoV19. Such events were acknowledged in the case whenever this information was available. These two circumstances would, however, lead to an overestimation of events linked to the ChadOx1-nCoV19 mass vaccination campaign. We did not have sufficiently detailed clinical notes to assess the diagnostic certainty of each case. However, patient notes were available and described symptomatology and other co-morbidities; these were used to assess the potential causes of the AESIs that showed an increase. Finally, although efforts were made to exclude all potential COVID-19 related healthcare visits from the analysis, residual cases classified in less obvious ICD categories, or undiagnosed COVID-19 may have interfered with the results.

## 5 Conclusion

A six-week follow-up of 77.683 adults vaccinated with ChadOx1-nCoV19 and a total of 151.734 doses administered did not result in any safety concerns. A slight increase in the number of ER visits was likely related to typical immunization events such as local pain or myalgia and to a decreased fear of being exposed to COVID-19 while visiting the health facilities. Of the 18 AESIs studied, only Bell’s palsy showed an increased number of cases that could not be attributed to other causes.

## Funding

This research was funded by the Brazilian Council for Scientific and Technological Development (CNPq), grant number 401575/2021-7, and the Bill and Melinda Gates Foundation (BMGF), grant number INV-017149.

## Declaration of Competing Interest

The authors declare that they have no known competing financial interests or personal relationships that could have appeared to influence the work reported in this paper.
